# Prevalence of smoking habits, attitudes and knowledge on counteractive strategies among a sample of healthcare employees: results of the smoking-free health environments project in the province of Palermo, Italy

**DOI:** 10.3389/fpubh.2024.1335937

**Published:** 2024-02-05

**Authors:** Claudio Costantino, Nicole Bonaccorso, Giuseppa Minutolo, Martina Sciortino, Giovanna Ripoli, Marco Berardi, Maurizio Gallo, Stefania Nastasi, Stefano Serra, Elisa Trapani, Eugenio Busalacchi, Provvidenza Ficano, Salvatore Siciliano, Palmira Immordino, Walter Mazzucco, Vincenzo Restivo, Francesco Vitale, Alessandra Casuccio

**Affiliations:** ^1^Department of Health Promotion Sciences, Maternal and Infant Care, Internal Medicine and Medical Specialties (PROMISE) “G. D’Alessandro”, University of Palermo, Palermo, Italy; ^2^Palermo Local Health Authority, Palermo, Italy; ^3^Dedalus Unit - Palermo Local Health Authority, Palermo, Italy; ^4^University Hospital - UH - “P. Giaccone” of Palermo, Palermo, Italy; ^5^“Villa Sofia-Cervello” Hospital of Palermo, Palermo, Italy; ^6^University of Enna “Kore”, Enna, Italy

**Keywords:** smoking, prevalence, smoking cessation, quit smoking, health facility

## Abstract

**Introduction:**

Tobacco use is responsible for over 7 million deaths annually, making smoking the leading cause of preventable mortality globally. Over the last two decades in Italy, the prevalence of smoking among physicians has consistently decreased, while it remains higher and is gradually decreasing among non-physician healthcare workers. The aim of this study was to investigate the Prevalence of smoking habits, attitudes, and knowledge on counteractive strategies among employees in the Primary Healthcare Facilities in the Province of Palermo, Italy.

**Methods:**

A cross-sectional survey was conducted between June 2020 and December 2020 through a previously validated anonymous questionnaire structured in four sections including 34 items. Data were analyzed using Stata/MP 12.1 statistical software.

**Results:**

Overall, 2,645 participants answered the questionnaire. The prevalence of either current or former smokers was 18.6%. Based on the multivariable analysis conducted, a significantly higher frequency of current smokers was observed among male participants (AdjOR: 1.29; CI95%: 1.02–1.64) and those belonging to the Surgical Unit (AdjOR: 1.92; CI95%: 1.27–2.90). Conversely, the prevalence of current smokers was significantly lower among those with at least one child (AdjOR: 0.67; CI95%: 0.49–0.91), with an educational qualification equal to or greater than a graduation degree (AdjOR: 0.56; CI95%: 0.43–0.73), those who considered second-hand smoke harmful (AdjOR: 0.06; CI95%: 0.008–0.60), those who had observed smoking or detected the smell of smoke in their workplace (AdjOR: 0.64; CI95%: 0.45–0.91). Furthermore, the prevalence of current smokers was significantly lower among participants who believed that healthcare professionals could play a crucial role in influencing their patients’ lifestyles (AdjOR: 0.67; CI95%: 0.50–0.90) and among those who recommend their patients to quit smoking (AdjOR: 0.35; CI95%: 0.24–0.51).

**Discussion:**

The results of the current research demonstrate that, despite the decline in smoking prevalence among physicians, the rate of smokers among healthcare facility employees remains unacceptably high. This underscores the need to re-evaluate current anti-tobacco strategies in the workplace.

## 1 Introduction

Tobacco use is responsible for over 7 million deaths annually, making smoking the leading cause of preventable mortality globally (1). It causes diseases such as lung cancer, chronic obstructive pulmonary disease and coronary heart disease (1, 2).

According to data from the Doxa-ISS survey, presented on World No Tobacco Day, May 31, 2022, there are 12.4 million smokers in Italy, constituting 24.2% of the population. Among them, 7.5 million are men (60%) and 4.9 million are women (40%) (3).

Quitting smoking enhances life expectancy and reduces the risk of chronic disease (4). The earlier an individual quits smoking, the greater the benefit. Studies in the United Kingdom and United States estimate that quitting in young adulthood can add an average of 10 years life expectancy (5). In the United States alone, economic losses due to cigarette smoking, which include both direct (healthcare) and indirect (the impact on productivity of diseases caused by smoking) costs, exceeded $891 billion in 2020, accounting for 4.3% of the US GDP (6). The economic loss is over 10 times higher than the $92 billion in revenue generated by the cigarette industry. It is particularly concerning, though not surprising, to note that some of the states with the highest economic losses have the weakest tobacco control policies (6).

Policies restricting indoor worksite tobacco use were implemented more than two decades ago. More recently, these policies have been expanded to include outdoors areas, with hospitals leading the trend by restricting smoking throughout their premises. This is due to the recognition that the harmful effects of smoking extend beyond the smoker, with environmental tobacco smoke causing over 1.2 million additional deaths annually among non-smokers (7, 8).

Environmental tobacco smoke (ETS) originates from the smoldering end of the tobacco product in between puffs, known as sidestream smoke (SS), and from the smoker’s exhaled smoke. Other contributors to ETS include minor amounts of smoke that escape during the puff-drawing from the burning cone and some vapor-phase components that diffuse through the cigarette paper into the environment. These various components are released into the environment and are diluted by ambient air. (9). Exposure to second-hand smoke involves the unintentional inhalation of carcinogens and other toxic components detrimental to health (10).

There is substantial scientific evidence indicating that second-hand smoke poses serious health risks to non-smokers. Non-smokers exposed to second-hand smoke are at risk of developing most of the same diseases that affect smokers (11).

According to the Environmental Protection Agency (EPA), exposure to environmental tobacco smoke is one of the most prevalent and hazardous indoor air pollutants (11).

The United States Surgeon General and the National Academy of Sciences have concluded that second-hand smoke can also induce lung cancer in smokers and children of smoking parents have a higher incidence of pneumonia, bronchitis, and asthma attacks compared to children of non-smoking parents (12).

In addition to respiratory diseases, passive smoking is also reported to increase the risk of coronary heart disease and heart attacks by 20%, primarily due to nicotine and carbon monoxide exposure (13).

Reducing the prevalence of tobacco use in general, is a paramount public health objective (14).

With the enactment of the “Protection of the Health of Non-Smokers” law in 2005 (January 16, 2003, n.3 art. 51), Italy became the first major European country to introduce legislation regulating smoking in all public and private indoor spaces, including workplaces and healthcare facilities. This law has been considered an effective model for public health interventions throughout Europe (15), and has been adopted by many other European countries and around the world, often with even more stringent regulations, such as the prohibition of smoking rooms (16).

According to data from the Italian surveillance system between 2014 and 2018, over the last two decades in Italy, the prevalence of smoking among health care professionals (HCPs) has consistently decreased, while it remains higher and is gradually decreasing among non-physician healthcare workers (17).

The work environment is an important place of exposure to active and passive smoking. In fact, the greatest number of smokers are concentrated in the 25–44-year age group, occupationally active individuals who spend most of their day in a work environment where they carry out their smoking habit.

The primary objective of this study is to investigate the Prevalence of smoking habits, attitudes, and knowledge on counteractive strategies among employees in the Primary Healthcare Facilities in the Province of Palermo, Italy. By profiling the tobacco habits of healthcare workers (HCWs) and providing essential information, we aim to contribute to a comprehensive understanding of the smoking landscape within healthcare settings. This study not only seeks to shed light on the current scenario but also aspires to serve as a foundation for targeted health promotion initiatives. Through this exploration, we intend to offer valuable insights that can guide the development of specific interventions to promote health, facilitate smoking cessation, and safeguard non-smokers from environmental tobacco exposure.

## 2 Materials and methods

“Smoke-Free Health Environments” is a project conducted by an inter-agency working group in the Province of Palermo, Italy. It was coordinated by the Local Health Unit and the Department of Health Promotion, Maternal and Child Health, Internal Medicine, and Specialties of Excellence “G. D’Alessandro” of the University of Palermo. The project was carried out among the personnel of two Palermo hospitals (“Villa Sofia-Cervello” Hospital, University Hospital (UH)—“P. Giaccone” of Palermo), as well as the Local Health Unit (LHU) of Palermo.

The “Smoke-Free Health Environments” project aims to promote health in the workplace and the community while extending protection from second-hand smoke within healthcare facilities.

The project is also part of the Sicilian regional prevention plan (National Health Plan 2016 Az. 4.1.1), which includes interventions aimed at healthcare professionals to promote healthy lifestyles and advise patients to quit smoking (18).

The target population of this study consists of employees from the four healthcare facilities described above.

The Palermo Metropolitan Area, formerly known as the province, is not only the most populous area in Sicily (the fourth most populous region in Italy) but also ranks as the fifth most populous province in Italy (19).

At the time of the questionnaire administration, the LHU of Palermo had 3,563 employees, the UH of Palermo had 2,323, and the Villa Sofia-Cervello Hospital had 1,213 employees. Subsequently, for those who voluntarily expressed the desire, workgroups were organized and led by the Local Health Unit’s addiction service in Palermo to facilitate smoking cessation.

### 2.1 Data collection

A cross-sectional survey was conducted to assess the prevalence, knowledge, and smoking habits among employees in Palermo’s healthcare facilities. The research team designed and validated a self-administered anonymous questionnaire to investigate their smoking habits.

Questionnaire administration occurred between June 2020 and December 2020 through dedicated links on the Google Modules^®^ platform. Each worker was granted access to the questionnaire through their individual, password-protected personal page on the healthcare facilities’ website. This approach ensured exclusive access for each worker, with the authentication process relying on personal passwords to prevent duplicate responses. The questionnaire was administered anonymously, and participants were provided with information about the study’s purpose, data handling methods, data protection, and then asked to provide informed consent.

One of the healthcare organizations involved in the project, Villa Sofia-Cervello Hospital, had previously revised its smoking policies in 2013 and provided employee training to raise awareness of this critical public health issue. To distinguish this healthcare organization from others, we introduced the “Educational Intervention” variable to further evaluate the effectiveness of the training intervention that took place before administering our questionnaire.

All data, once the questionnaires were completed, were automatically recorded in a password-protected Excel file (1997–2003) accessible only to the Working Group to ensure privacy. The study received prior approval from the Ethics Committee at the University Hospital “P. Giaccone” of Palermo during meeting n.03/2019 on March 20, 2019.

### 2.2 Questionnaire structure

The questionnaire’s reliability and validity were assessed in a preliminary pilot testing study with 30 healthcare workers. In this study, Cronbach’s alpha was calculated, yielding a satisfactory reliability coefficient of 0.78. The questionnaire took the 30 healthcare workers about 10 min.

The questionnaire comprises 34 items divided into four sections, intended to investigate:

- Socio-demographic aspects: age, gender, residence and work location;
- Smoking habits at home and in the workplace;
- Knowledge of current smoking regulation legislation;
- Willingness to quit smoking and the potential supports used for cessation;
- Knowledge and perceptions regarding second-hand smoke exposure;
- Opinion on the role played by healthcare professionals in influencing their patients’ lifestyle;
- Perception on the need to improve stricter enforcement of smoke-free legislation and the necessity of implementing smoking areas in outdoor spaces.

Smoking status was determined following Centers for Disease Control and Prevention (CDC) guidelines (20) and categorized into three groups:

- *Current smoker*: An adult who has smoked 100 cigarettes in his or her lifetime and currently smokes.
- *Former smoker*: An adult who has smoked at least 100 cigarettes in his or her lifetime but who had quit smoking at the time of interview.
- *Never smoker*: An adult who has never smoked, or who has smoked less than 100 cigarettes in his or her lifetime.

### 2.3 Statistical analysis

Data obtained were collected in a database in Microsoft Excel format (which were also automatically generated by the Google^®^ Modules online questionnaire administration system) and subsequently analyzed through the statistical software package Stata/MP 12.1 (StataCorp LP, College Station, TX, United States).

Absolute and relative frequencies were calculated for categorical (qualitative) variables. Differences in qualitative variables were analyzed using chi-square tests (or Fisher’s exact test when appropriate). The chosen level of statistical significance was a value of *p* less than 0.05.

In the univariate and multivariate analyses, individuals identified as current smokers were categorized as ‘Yes’, while those classified as former smokers and non-smokers were categorized as ‘No’. This classification allowed for a nuanced exploration of the factors associated with current smoking habits, providing valuable insights into the determinants of smoking prevalence within our study cohort.

All variables showing a statistically significant association with a higher prevalence of tobacco use in the univariate analysis were included in a multivariate backward stepwise logistic regression model. Furthermore, all variables with a value of *p* ≤ 0.20 were included in the multivariate model to ensure a more conservative approach. Crude odds ratios (ORs) and adjusted odds ratios (adj-ORs) with their 95% confidence intervals (CIs) were calculated.

## 3 Results

The total number of participants in the survey was 2,645 (*n* = 1,360 females, *n* = 1,285 males).

The majority of respondents worked at the University Hospital “P. Giaccone” of Palermo (45.7%; *n* = 1,208), 40.7% (*n* = 1,076) in the Local Health Unit of Palermo and 13.6% (*n* = 361) at Villa Sofia-Cervello Hospital.

The overall response rates were 37.2, 52.1% for the UH of Palermo, 30.2% at the LHU of Palermo and 29.8% at the “Villa Sofia-Cervello” Hospital.

The distribution of respondents by age group showed a prevalence of the 50–59 years of age class (37.2%; *n* = 984), followed by those over 60 years of age (24.1%; *n* = 639), participants aged 40–49 years of age (22.1%; *n* = 585), 30–39 years of age (12.7%; *n* = 335), and those aged 29 years or younger (3.9%; *n* = 102).

Seventy-five percent of respondents (*n* = 1989) were married or in a *de facto* relationship’, while 25% (*n* = 656) were divorced, widowed, or single.

Additionally, 74.7% of the enrolled participants reported having at least one child, as opposed to the remaining 25.3% of them (Table 1).

**Table 1:** Socio-demographic characteristics and work location (*n* = 2,645).

	n (%)
Gender	
Male	1,285 (48.6)
Female	1,360 (51.4)
Workplace	
“Villa Sofia-Cervello” Hospital	361 (13.6)
University Hospital “P. Giaccone” of Palermo	1,208 (45.7)
Local Health Unit of Palermo	1,076 (40.7)
Age group	
19-29	102 (3.9)
30-39	335 (12.7)
40-49	585 (22.1)
50-59	984 (37.2)
60+	639 (24.1)
Civil status	
Married/ in a *de facto* relationship’	1,989 (75)
Divorced/widowed/single	656 (25)
Children	
At least one	1,977 (74.7)
No one	668 (25.3)
Level of education	
Primary/Secondary /High school license	883 (33.4)
Graduation/Post graduate	1,762 (66.6)
Operating unit	
Medical Unit	1,016 (38.4)
Diagnostic/Public health Unit	651 (24.6)
Surgical Unit	387 (14.6)
Administrative/other units	591 (22.4)
Role in healthcare company	
Medical doctor	765 (28.9)
Non-medical healthcare executive	160 (6)
Nurse	684 (25.9)
Auxiliary Health Care Assistant	20 (0.8)
Other healthcare professions	364 (13.8)
Administrative/other units	652 (24,6)

Regarding the educational level, 66.6% of respondents had a degree equal to or greater than a university degree, followed by 33.4% of participants with a Primary/Secondary school or High school diploma. Most of the sample interviewed belonged to a Medical Unit (38.4%), 24.6% to Diagnostic/Public health Unit, 14.6% to Surgical Unit and 22.4% were administrative technical staff or affiliated with other units.

Furthermore, 28.9% (*n* = 772) of respondents were a medical doctor, 25.9% were nurses, 24.6% were administrative/other staff, while 13.8% of participants were other healthcare professionals (Table 1).

In Table 2, prevalence, knowledge, and smoking habits were reported. Eighteen percent of the sample (*n* = 492) stated they were current smokers, while 63% of respondents were non-smokers (*n* = 1,662) or former smokers (19%; *n* = 491).

**Table 2:** Prevalence, knowledge and smoking habits (*n* = 2,645).

	n (%)
Smoking habit	
Current Smokers	492 (18.6)
Former-smokers	491 (18.6)
Never-smokers	1,662 (62.8)
Consider exposure to second-hand smoke harmful	
Yes	2,290 (98.2)
No	17 (0.7)
Do not know	25 (1.1)
Ever observed someone smoke or smell of smoke in workplace	
No, never	1,412 (58.3)
Yes	1,011 (41.7)
Role of HCPs in influencing the lifestyles of their patients	
Yes	1,961 (80.9)
No	462 (19.1)
Favorable to a dedicated outdoor space for smokers	
Yes	1,899 (77.3)
No	557 (22.7)

Almost all the sample considered exposure to second-hand smoke harmful (98.2%; *n* = 2,290) and 58% of the respondents reported that they had never observed anyone smoking or detected the smell of smoke in their workplace.

A large majority of those interviewed (80.9%; *n* = 1961) believe that HCPs can influence their patients’ lifestyles, and 68.5% of HCPs, claimed to have advised their patients to quit smoking (in both cases this is observed more frequently in non-smokers). Over three quarters (77.3%) of the sample expressed support for the establishment of a dedicated outdoor space for smokers on company premises.

In Table 3, univariate and multivariate analysis of factors associated with current smokers were reported. Multivariate analysis revealed a significantly higher frequency of current smokers among male participants (AdjOR:1.31; CI95%:1.03–1.67) and those belonging to the Surgical Unit (AdjOR:2.12; CI95%:1.37–3.28). Conversely, the prevalence of current smokers was significantly lower among those with at least one child (AdjOR:0.67; CI95%:0.49–0.91), with an educational qualification equal to or greater than a graduation degree (AdjOR:0.58; CI95%:0.43–0.78), those who considered second-hand smoke harmful (AdjOR:0.07; CI95%:0.01–0.60) and those who had observed smoking or detected the smell of smoke in their workplace (AdjOR:0.65; CI95%:0.46–0.92). Furthermore, the prevalence of current smokers was significantly lower among participants who believed that healthcare professionals could play a crucial role in influencing their patients’ lifestyles (AdjOR:0.66; CI95%:0.49–0.89) and among those who recommend their patients to quit smoking (AdjOR:0.36; CI95%:0.25–0.53; Table 3).

**Table 3:** Factors associated with higher prevalence of tobacco use at uni (Crude OR) and multivariate (Adjusted OR) analyses. (95% CI: 95% confidence interval).

	Current smokers (Yes vs. No)			
	Crude OR (95% CIs)	Value of *p*	Adj-OR (95% CIs)	Value of *p*
Gender				
Female	Reference	0.01	Reference	0.02
Male	1.32 (1.08–1.61)		1.31 (1.03–1.67)	
Age classes				
≥ 50 years	Reference	0.49		
≤ 49 years	0.93 (0.76–1.14)			
Civil status				
Married/cohabiting	Reference	0.05	Reference	0.08
Divorced/widowed/single	1.41 (1.13–1.75)		1.29 (0.96–1.74)	
Children				
No one	Reference	0.04	Reference	0.01
At least one	0.75 (0.61–0.94)		0.67 (0.49–0.91)	
Level of education				
Primary/Secondary/High school license	Reference	0.001	Reference	0.001
Graduation/Post graduate	0.55 (0.45–0.68)		0.58 (0.43–0.78)	
Operating unit				
Administrative/other units	Reference		Reference	
Surgical Unit	1.13 (0.84–1.54)	0.4	2.12 (1.37–3.28)	0.001
Diagnostic/Public health Unit	0.68 (0.51–0.91)	0.05	1.07 (0.73–1.58)	0.70
Medical Unit	0.72 (0.56–0.93)	0.04	1.43 (0.97–2.09)	0.06
Role in healthcare company				
Medical doctor	Reference		Reference	
Nurse	1.10 (0.83–1.46)	0.4	1 (0.70–1.43)	0.9
Other	1.48 (1.16–1.88)	0.001	1.20 (0.85–1.68)	0.2
Working in facilities with educational intervention				
No	Reference	0.01	Reference	0.02
Yes	0.67 (0.49–0.92)		0.66 (0.47–0.95)	
Consider exposure to second-hand smoke harmful				
No	Reference	0.001	Reference	0.01
Yes	0.09 (0.03–0.27)		0.07 (0.01–0.60)	
Ever seen someone smoke or smell of smoke in work place				
No	Reference	0.001	Reference	0.01
Yes	0.59 (0.48–0.74)		0.65 (0.46–0.92)	
Role of HCPs in influencing the lifestyles of their patients				
No	Reference	0.001	Reference	0.008
Yes	0.53 (0.42–0.67)		0.66 (0.49–0.89)	
Advising their patients to quit smoking				
No	Reference	0.001	Reference	0.001
Yes	0.32 (0.23–0.43)		0.36 (0.25–0.53)	
Visible “no smoking” sign				
No	Reference	0.001	Reference	0.15
Yes	1.71 (1.25–2.34)		1.39 (0.88–2.22)	
Identification of the person responsible for monitoring compliance with the no smoking order				
No	Reference	0.001	Reference	0.17
Yes	1.41 (1.12–1.78)		1.21 (0.91–1.62)	

Finally, healthcare personnel who attended the educational intervention (which, as previously mentioned, was conducted only at the Villa Sofia-Cervello Hospital) exhibited a significantly lower prevalence of current smokers (AdjOR:0.66; CI95%:0.47–0.95).

## 4 Discussion

Smoking is still a major public health problem. This study aimed to investigate prevalence, knowledge and habits related to smoking among employees of healthcare facilities in the Province of Palermo (21). The prevalence of current smokers in our sample is lower than Italian surveillance data on HCPs between 2014 and 2018 (18.6% vs. 23.0%) (22). According to this surveillance, the major predictive factors to be a current smoker is nearly 2-fold in medical doctors aged under 35 years old, belonging to male sex, and living in Southern Italy. On the other hand, both worsened financial situation and lower educational level was associated with other HCPs categories (22). The association between male sex and current smoking is confirmed in this study, while having a higher educational level plays a protective role against smoking, as well as having almost a child. A higher prevalence of current smokers among HCPs was found also in Palestina, which was nearly twice that obtained in this study (34.5% vs. 18.6%) (23). Furthermore, male sex was 7-fold associated with current smoker status (23).

Surgical facilities increase the odds to be a current smoker twice, probably due to working conditions leading to a higher level of stress (24). Workplaces and related working networks can influence HCPs’ behavior toward tobacco smoke. According to Evenhuis et al., HCPs with relevant level of working-related stress seem to be a higher predisposition to smoke than others. (25). Smoking habit is reinforced in stressed participants following triggering events, even in who had quit previously (26). In detail, the odds to smoke again after quitting is over 3-fold higher in participants under stress than others (26). In some way, HCPs can also be considered disadvantaged due to their cumulative risk factors for smoking. They often contend with heavy workloads, working over 50 h a week, and frequently working night shifts, especially during the recent SARS CoV-2 pandemic (27–29). Prolonged high levels of stress can result in adverse health outcomes when left unaddressed and can significantly contribute to smoking habits (30). Excessive stress can even lead to depressive disorders and an increased risk of suicides, that are also risk factors for smoking (31, 32). Notably, existing literature consistently indicates that disadvantaged populations exhibit higher smoking rates compared to others (30).

As health experts and promoters, HCPs have a central role to play in reducing the global tobacco epidemic (33). HCPs are essential weapons to promote smoking cessation and treat tobacco dependence in their patients, following evidence-based tobacco cessation guidelines (34, 35). The WHO Framework Convention on Tobacco Control (FCTC) emphasizes the importance of HCPs setting an example by not using tobacco (36). In our study, HCPs who are current smokers have a reduced odds of advising the own patients to quit smoking and perceiving the impact of their lifestyles on the own patients. According to Mizher et al., current smokers’ status among HCPs reduced the effectiveness of quitting smoking interventions in patients of 9.3–13.0% (23), confirming our results. Working in healthcare facilities with Educational Intervention can be considered a protective factor against tobacco smoke. On the other hand, smoking ban sign could have an impact on smoking status. A literature scoping review have shown tobacco smoke use was declined following a stricter smoking prohibition in workplaces (25). However, in this study the prevalence of current smokers does not change after visible sign “no smoking.”

Despite the key role that HCPs, in particular physicians, in the fight against smoking, as highlighted in our study, it is alarming that many of them continue to smoke. This is surprising, given that they should serve as role models for their patients and be well-informed about the health risks associated with tobacco (37), especially non-communicable diseases such as cardiovascular diseases and cancer (1, 2). On the contrary, they represent a bad model for the own patients, reducing the effectiveness of quitting smoking interventions (23).

Despite these challenges, two important findings from several studies have emerged. Health professionals have greater success in convincing patients to quit smoking if they are non-smokers or former smokers (37, 38). The results of our survey suggest that HCPs are aware of their role in influencing their patients’ lifestyles, which is encouraging for the design of effective strategies to reduce the risk of tobacco smoke in healthcare settings.

Furthermore, current smokers who seek support and advice from their healthcare providers have a better chance of quitting than those who attempt to quit on their own (39–41).

Additionally, our study indicates that a lower prevalence of smoking habits is observed in the healthcare organization that implemented anti-smoking education interventions prior to the administration of our questionnaire (AOR Villa Sofia-Cervello). This suggests that investing in campaigns to promote healthy lifestyles is one of the best strategies to combat this phenomenon.

Given that HCPs are seen as examples to the community and their colleagues, it is imperative that hospitals represent a place where a culture of health promotion and smoking cessation is fostered (42, 43). To achieve this goal, it is essential that all employees of healthcare facilities recognize their vital yet indispensable role in this regard, not only to protect each patient who accesses a health facility, but also to serve as virtuous model to be emulated.

In this context, smoking cessation assistance services could be structured to offer employees free or reduced-cost prescribed medications. Previous research has found that employees are more likely to enroll in a smoking cessation program if the employer covers all costs (44).

Recognizing the fundamental role of Primary Healthcare (PHC) becomes imperative in this effort. Unfortunately, in Sicily, PHC has historically been underdeveloped, with past Regional Governments not prioritizing its significance. This lack of consideration on PHC represents a significant limitation in our healthcare system compared to other international contexts (45). Improving the integration and prioritization of PHC by Regional Healthcare authorities is essential, especially considering its key role in preventative strategies against smoking and other health challenges.

The findings of this study reveal that attitudes and knowledge regarding smoking are significantly more favorable among the staff at Villa Sofia-Cervello Healthcare Facility. This improvement could be probably attributed to the educational interventions conducted among HCPs of Villa Sofia-Cervello in the previous years. This insight suggests the importance of standardizing targeted educational interventions for healthcare professionals across all facilities. Such interventions would not only promote greater awareness of the issue but could also contribute to a reduction in smoking-related habits. This recommendation aligns directly with the primary objectives of the Smoking-Free Health Environments project, emphasizing the significance of educational initiatives to foster healthier work environments and decrease tobacco consumption among healthcare professionals.

Successful strategies to reduce tobacco use and mitigate its environmental harms must become a priority for all the countries. Achieving this success will require innovative and fundamentally different approaches, including a diverse mix of ideas and new partnerships, particularly within hospitals, where a culture of public health and smoking cessation must be promoted.

There are some limitations of the present study that need to be highlighted. Firstly, the limited number of participants may lead to a lack of representativeness, especially for some categories of HCPs. Also, COVID-19 could play a negative role in the recruitment of HCPs, specifically for that categories involved in the first line contrast of the pandemic Health Emergency (46). Secondly the surveys were primarily conducted through online recruitment, and the fact that participants were voluntary may introduce potential selection bias. Nevertheless, the strategy used for questionnaire administration (before viewing the payslip) and the good response rate observed (over 30% in all healthcare facilities and over 35% overall) may have helped mitigate the selection bias.

## 5 Conclusion

The results of the current research demonstrate that, despite the decline in smoking prevalence among physicians, the rate of smokers among healthcare facility employees remains unacceptably high. This underscores the need to re-evaluate current anti-tobacco strategies in the workplace. Understanding the factors associated with tobacco smoke could help to realize focused interventions in improving knowledge and the awareness of negative effects of smoke on human health. Further research should be done to evaluate the effectiveness of such interventions to remove risk factor for tobacco smoke.

It is considered essential to intervene in order to raise awareness among healthcare company employees about the risks of smoking and the pivotal role they can play, not only in safeguarding each patient accessing a healthcare facility but also in serving as a virtuous model to be emulated. This emphasizes the need to encourage responsible behaviors, especially within healthcare environments. Furthermore, more rigorous studies are required to assess the impact of expanded outdoor smoke-free boundaries on smoking behavior. New stricter policies against tobacco smoke in healthcare facilities and in other indoor environments should be considered and their effectiveness requires dedicated future studies. Additional research should also examine the relationship between smoking cessation and the provision of tobacco treatment services, determining the optimal levels of services necessary to assist employees in their tobacco cessation efforts.

## Data availability statement

The raw data supporting the conclusions of this article will be made available by the authors, ensuring the anonymity of the respondents to the questionnaire, without undue reservation.

## Ethics statement

The studies involving humans were approved by Comitato Etico Palermo 1 Ethics Committee at the University Hospital “P. Giaccone” of Palermo. The studies were conducted in accordance with the local legislation and institutional requirements. The participants provided their written informed consent to participate in this study.

## Author contributions

CC: Conceptualization, Data curation, Formal analysis, Funding acquisition, Methodology, Visualization, Writing – original draft. NB: Data curation, Formal analysis, Methodology, Validation, Visualization, Writing – original draft. GM: Data curation, Investigation, Methodology, Resources, Software, Validation, Writing – review & editing. MS: Investigation, Methodology, Resources, Validation, Writing – review & editing. GR: Investigation, Resources, Validation, Writing – review & editing. MB: Investigation, Resources, Validation, Writing – review & editing. MG: Investigation, Resources, Validation, Writing – review & editing. SN: Investigation, Resources, Validation, Writing – review & editing. StS: Investigation, Resources, Validation, Writing – review & editing. ET: Investigation, Resources, Validation, Writing – review & editing. EB: Investigation, Resources, Validation, Writing – review & editing. PF: Investigation, Resources, Validation, Writing – review & editing. SaS: Resources, Validation, Writing – review & editing, Investigation. PI: Data curation, Visualization, Writing – review & editing. WM: Conceptualization, Data curation, Formal analysis, Supervision, Writing – review & editing. VR: Conceptualization, Data curation, Software, Writing – review & editing. FV: Data curation, Formal analysis, Funding acquisition, Project administration, Supervision, Writing – review & editing. AC: Conceptualization, Data curation, Formal analysis, Funding acquisition, Methodology, Project administration, Supervision, Writing – original draft.
